# Changes of gut microbiota and tricarboxylic acid metabolites may be helpful in early diagnosis of necrotizing enterocolitis: A pilot study

**DOI:** 10.3389/fmicb.2023.1119981

**Published:** 2023-03-15

**Authors:** Ting-Ting Du, Xiao-Chen Liu, Yu He, Xiong Gao, Zhen-Zhen Liu, Zheng-Li Wang, Lu-Quan Li

**Affiliations:** ^1^Neonatal Diagnosis and Treatment Centre of Children’s Hospital of Chongqing Medical University, National Clinical Research Center for Child Health and Disorders, Ministry of Education Key Laboratory of Child Development and Disorders, International Science and Technology Cooperation Base of Child Development and Critical Disorders, Chongqing Key Laboratory of Pediatrics, Chongqing, China; ^2^Jiangxi Hospital Affiliated Children’s Hospital of Chongqing Medical University, Nanchang, China

**Keywords:** gut microbiota, succinate, tricarboxylic acid metabolites, NEC, early diagnosis

## Abstract

**Purpose:**

We aimed to explore the value of gut microbiota and tricarboxylic acid (TCA) metabolites in early diagnosis of necrotizing enterocolitis (NEC) among infants with abdominal manifestations.

**Methods:**

Thirty-two preterm infants with abdominal manifestations at gestational age ≤ 34 weeks were included in the study and were divided into non-NEC (*n* = 16) and NEC (*n* = 16) groups. Faecal samples were collected when the infants were enrolled. The gut microbiota was analysed with high-throughput sequencing, and TCA metabolites were measured with multiple reaction monitoring (MRM) targeted metabolomics. Receiver operating characteristic (ROC) curves were generated to explore the predictive value of the obtained data.

**Results:**

There was no significant difference in alpha diversity or beta diversity between the two groups (*p* > 0.05). At the phylum level, *Proteobacteria* increased, and *Actinomycetota* decreased in the NEC group (*p* < 0.05). At the genus level, *Bifidobacterium* and *Lactobacillaceae* decreased significantly, and at the species level, *unclassified Staphylococcus*, *Lactobacillaceae* and *Bifidobacterium animalis subsp. lactis* decreased in the NEC group (*p* < 0.05). Further Linear discriminant analysis effect sizes (*LEfSe*) analysis showed that the change in *Proteobacteria* at the phylum level and *Lactobacillaceae* and *Bifidobacterium* at the genus level scored higher than 4. The concentrations of succinate, L-malic acid and oxaloacetate in the NEC group significantly increased (*p* < 0.05), and the areas under the ROC curve for these metabolites were 0.6641, 0.7617, and 0.7344, respectively.

**Conclusion:**

Decreased *unclassified Staphylococcus*, *Lactobacillaceae* and *Bifidobacterium animalis subsp. lactis* at the species level as well as the increase in the contents of some TCA metabolites, including succinate, L-malic acid and oxaloacetate, have potential value for the early diagnosis of NEC.

## 1. Introduction

Necrotizing enterocolitis (NEC) is a devastating gastrointestinal disease in the neonatal period, especially in preterm infants (Neu, 2020). According to the multicenter studies, the incidence rate of NEC in preterm infants is reported to be 3–12% (Eaton, 2017; Meister et al., 2020; Cao et al., 2021) and the mortality is 9.4–17.6% (D'Apremont et al., 2020; Cao et al., 2022; Xu et al., 2022) and can be much higher in those requiring surgeries (Geng et al., 2018; D'Apremont et al., 2020). Moreover, among survivors, complications such as intestinal stricture, short bowel syndrome and delayed neurological development seriously affect their quality of life (Frost et al., 2017; Berken and Chang, 2022). Therefore, early diagnosis of NEC will help decrease mortality and improve the outcome of NEC (Neu, 2020).

At present, the diagnosis of NEC is mainly based on clinical manifestations such as bloody stools, vomiting, diarrhea, abdominal distension, and imaging presentation of pneumatosis intestinalis, portal venous gas or pneumoperitoneum (Neu, 2020). However, in preterm infants with lower gestational age, typical imaging presentation is easily missed (Sharma and Hudak, 2013; Yu et al., 2016). Thus, biomarkers based on etiology and pathogenesis would be helpful in the early diagnosis of NEC among infants with abdominal manifestations (Ng, 2018).

The pathogenesis of NEC is not completely clear, and abnormal microbial colonization is thought to play an important role in the development of NEC (Neu, 2020). Dysbiosis of the gut microbiota may trigger NEC development, since germ-free mice do not develop NEC (Cassir et al., 2016). Numerous studies have shown that alterations in the gut microbiota occur prior to the onset of NEC (Pammi et al., 2017; Baranowski and Claud, 2019; Aziz et al., 2022), including decreased diversity, enrichment of *Proteobacteria*, and corresponding underrepresentation of *Firmicutes* and *Bacteroidota* (Warner and Tarr, 2016; Pammi et al., 2017). Partial gut microbiota changes, such as *Klebsiella*, *Clostridium*, *Clostridium perfringens* and *Propionibacterium* are regarded as potential biomarkers to early diagnosis of NEC (Morrow et al., 2013; McMurtry et al., 2015; Sim et al., 2015; Olm et al., 2019).

Altered gut microbiota may lead to changes in the produced metabolites such as bile acids, short-chain fatty acids (SCFAs), branched-chain amino acids, trimethylamine oxide, tryptophan and indole derivatives (McCarville et al., 2020; Agus et al., 2021) and some metabolites have been reported as bridge between microbiota and the development of diseases in human beings (Macia et al., 2015; Zhu et al., 2016). For example, excessive trimethylamine oxide, as production of intestinal microbiota fermenting the choline in food may cause the reverse transport of cholesterol and leads to atherosclerosis (Zhu et al., 2016). Bile acid produced by gut microbiota may lead to changes of interleukin-22 and then cause insulin resistance and infertility (Qi et al., 2019). High level of branched-chain amino acids could attenuate the inflammatory in Parkinson’s disease mice and reverse motor and non-motor dysfunctions and dopaminergic neuron impairment (Yan et al., 2022). Moreover, microbial metabolites have also been recognized as potential biomarkers of diseases (Nicholson et al., 2012; Sharon et al., 2014). SCFAs are used as markers of obesity, inflammatory bowel disease, non-alcoholic fatty liver disease, insulin resistance and type 2 diabetes mellitus (Canfora et al., 2019; Dong et al., 2019). One study found that SCFAs were already changed before the onset of NEC and may be help to early diagnose of NEC (Liu et al., 2022). Thus, early changes of microbiota and metabolites perhaps are helpful in early diagnosis of NEC in preterm infants.

Some of intermediates of the tricarboxylic acid (TCA) cycle such as succinate, malic acid and oxaloacetate are not only the metabolites of cellular metabolism (Akram, 2014), but also as intermediates of bacterial fermentation (Louis and Flint, 2017). Previous study has showed that fumarate, citrate and malic acid were positively correlated with *Eubacterium coprostanoligenes group*, *Faecalibacterium*, and *Oscillibacter* (Xie et al., 2022) and succinate, which is known as an intermediate metabolite in bacterial fermentation for the production of propionate, can be produced by *Bacteroidota* and *Prevotella* (Xu et al., 2003; Kovatcheva-Datchary et al., 2015). Thus, the change of these metabolites may regulate intestinal inflammation and immune response (Connors et al., 2018; Macias-Ceja et al., 2019). Some intestinal TCA intermediates might be potential markers for early diagnosis of intestinal disease and host cardiometabolic health (Han et al., 2018; Wan et al., 2020). However, it remains unclear whether TCA metabolites might be useful to early diagnosis of NEC.

The aim of this study was to clarify whether the gut microbiota and its metabolites contribute to the early diagnosis of NEC among those infants with abdominal distention or vomiting, diarrhoea or bloody stools.

## 2. Subjects and methods

A prospective cohort study was performed in Children’s Hospital of Chongqing Medical University from April to November in 2021. This study was approved by the Ethics Committee of the Children’s Hospital of Chongqing Medical University (No. 2021.23) and registered in the China Clinical Trial Canter (ChiCTR2100044842). Parents of the enrolled infants all signed an informed consent form, and all infants were treated according to conventional treatment without any extra interventions.

### 2.1. Inclusion criteria

Infants who met the following criteria were enrolled: (1) infants with gestational age ≤ 34 weeks and (2) infants with one of the following manifestations including abdominal distention, vomiting, diarrhoea or bloody stools.

### 2.2. Exclusion criteria

The exclusion criteria were as follows: (1) infants who died during hospitalization for causes other than NEC, (2) infants with congenital gastrointestinal malformations (e.g., congenital intestinal atresia, megacolon, intestinal malrotation, etc.), (3) infants who were discharged against medical service, (4) infants who did not complete the determination of gut microbiota and metabolites, and (5) infants whose parents refused to participate in this study.

### 2.3. Grouping

Neonates were divided into NEC and non-NEC groups according to the final diagnosis. Infants in the NEC group were required to meet Bell’s diagnostic criteria (stage II or above) (Kliegman and Walsh, 1987). The non-NEC infants enrolled at the same time were matched 1: 1 according to gestational age and birth weight. The gestational age difference was less than 1 week, and the birth weight difference was less than 250 grams.

### 2.4. Faecal sample and clinical data collection

Naturally excreted faecal samples from the enrolled infants were collected with disposable sterile swabs on the day of enrolment and stored in the refrigerator at −80 degrees for further examination. Clinical data such as gender, gestational age, birth weight, feeding strategy, maternal condition and treatments in hospital duration were routinely collected. All infants enrolled were followed up until they were diagnosed with NEC or discharged from the hospital.

### 2.5. DNA extraction, PCR amplification and high-throughput sequencing

Half of each sample were used for the determination of microbiota and sequencing was performed with the standard kit by Applied Protein Technology Co., Ltd. (Shanghai, China) according to the standard protocol. Total genome DNA from samples was extracted using CTAB method and the concentration and purity was monitored on 1% agarose gels. According to the concentration, DNA was diluted to 1 ng/μl using sterile water. 16S rRNA genes were amplified used the specific primer 341F (CCTAYGGGRBGCASCAG) and 806R (GGACTACNNGGGTATCTAAT) to amplify the V3-V4 region (Zhang et al., 2020). All PCR reactions were carried out in 30 μL reactions with 15 μL of Phusion®High-Fidelity PCR Master Mix (New England Biolabs); 0.2 μM of forward and reverse primers, and about 10 ng template DNA. After initial denaturation for 1 min at 98°C, the cycle was as follows: (1) denaturation for 10 s at 98°C, (2) annealing for 30 s at 50°C, and (3) extension for 30 s at 72°C. The steps were performed for 30 cycles, with a final extension for 5 min at 72°C. PCR products were mixed with same volume of 1X loading buffer (contained SYB green) and purified with electrophoresis on 2% agarose gel. Samples with bright main strip between 400 and 450 bp were further purified with AxyPrepDNA Gel Extraction Kit (AXYGEN). Sequencing libraries were constructed using the NEB Next®Ultra™DNA Library Prep Kit for Illumina (NEB, USA) and the library quality was assessed on the Qubit@ 2.0 Fluorometer (Thermo Scientific) and Agilent Bioanalyzer 2100 system. At last, the library was sequenced on an Illumina Novaseq 6000 platform compared against the Silva reference database (Quast et al., 2013) and 250 bp paired-end reads were generated (Di Segni et al., 2018; Shahi et al., 2019).

### 2.6. Metabolite determination

The rest of same sample were used for the metabolite determination. 100 μL aliquots were mixed with 400 μL of cold methanol/acetonitrile (1: 1, v/v) at 4°C to remove the protein. The mixture was centrifuged for 20 min (14,000× *g*, 4°C). The supernatant was dried in a vacuum centrifuge and in the liquid chromatograph mass spectrometer (LC–MS) analysis, the dried supernatant was re-dissolved in 100 μL acetonitrile/water (1: 1, v/v), adequately vortexed, and then centrifuged (14,000× *g*, 4°C, 15 min) (Roca et al., 2021). The samples were placed in a 4°C autosampler with a column temperature of 45°C, a flow rate of 300 μL/min and an injection volume of 2 μL for separation in Agilent 1290 Infinity UHPLC (Agilent) with 10 mM ammonium acetate aqueous solution for mobile phase A and acetonitrile for mobile phase B. Mobile phase B changed linearly from 90 to 40% in 18 min and was maintained at 90% for 5 min. A 5500 QTRAP mass spectrometer (AB SCIEX, Massachusetts USA) was used for mass spectrometric analysis, and Multiquanta software was used for the extraction of peak area and retention time data. The retention time was corrected by standard energy metabolites for identification (Karu et al., 2018).

### 2.7. Data analysis

All data of clinical recorders and metabolites were analysed with SPSS statistical software (version 23; Chicago, USA). Quantitative data with a normal distribution are expressed as the mean ± standard deviation (SD) and were analysed with a *correlation t test*. Quantitative data from nonnormal distributions were expressed as interquartile ranges (IQRs) and analysed with the *Wilcoxon signed rank sum test*. Qualitative data were analysed with *Fisher’s exact test*. Reads were clustered into operational taxonomic units (OTUs) at 97% similarity (Rognes et al., 2016). A core curve and a rarefaction curve were performed to determine whether the sample size and sequences measured were sufficient. Analysis of similarities (ANOSIM) based on the Bray–Curtis distance was performed to determine whether the grouping was meaningful with the comparison of differences between groups and within groups (Clarke, 1993). The beta diversity was calculated with QIIME (version 1.9.1; Colorado, USA) and analysed with nonmetric multidimensional scaling analysis (NMDS) based on multidimensional spatial sample localization, analysis and categorization in R language (version 3.3.1; Auckland, New Zealand). Comparisons of gut microbiota composition were performed with the *Wilcoxon rank sum test*. Linear discriminant analysis effect sizes (*LEfSe*) obtained by the *Kruskal–Wallis rank sum test* were used to further characterize the variation among microbiomes (Segata et al., 2011). A correlation heatmap analysis was performed to show the relationship between metabolites and gut microbiota based on *Spearman rank correlation* in R language. Receiver operating characteristic (ROC) curves and all data were generated with GraphPad Prism (version 9.0; California, USA).

## 3. Results

### 3.1. Clinical information

**Figure 1:**
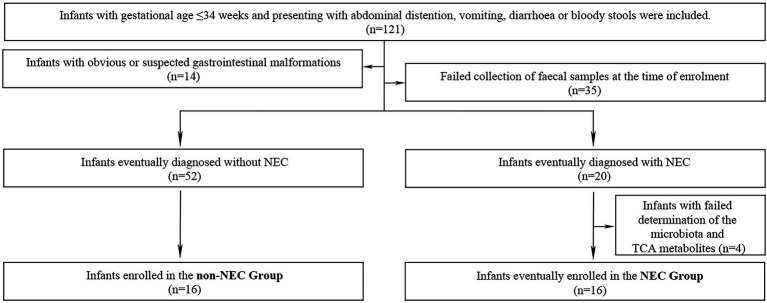
Inclusion, exclusion and grouping process in this study.

A total of 121 preterm infants were enrolled during the primary study period. Forty-nine infants were excluded due to obvious or suspected gastrointestinal malformations (*n* = 14), failure to collect faecal samples (*n* = 35). Other 4 infants were also excluded from further study because of failure to complete determination of the microbiota and metabolites (*n* = 4) in NEC infants. Therefore, 16 infants were enrolled in the NEC group and the other 16 infants without NEC were matched as the control group. The flow chart of this study was shown in Figure 1. The infants in the NEC group developed into NEC after 4.00 (0.00, 11.5) days after enrolment. There were no significant differences in general information and prenatal and postnatal risk factors of NEC and clinical manifestations when enrolled (*p* > 0.05). Infants in the NEC group had a longer hospital duration and a higher incidence of sepsis than those in the non-NEC group (*p* < 0.05, Table 1).

**Table 1:** Clinical features of infants enrolled in this study.

	Non-NEC (*n* = 16)	NEC (*n* = 16)	*X^2^/Z/t*	*P*
*General information*				
Male, % (*n*)	56.3 (9)	81.3 (13)	/	0.133
Gestational age, *x* ± S. D, w	30.47 ± 1.99	30.53 ± 2.33	0.231	0.820
Birth weight, *x* ± S. D, g	1464.69 ± 343.82	1440.31 ± 487.96	0.235	0.817
Caesarean section, % (*n*)	56.3 (9)	62.5 (10)	/	0.723
Apgar 1 min, *M* (P_25_–P_75_)	8 (5.25–9)	8 (7–9)	−0.079	0.937
Apgar 5 min, *M* (P_25_–P_75_)	9 (8.25–10)	9 (9–9)	−0.46	0.963
Apgar 10 min, *M* (P_25_–P_75_)	10 (9–10)	9 (9–9)	−1.098	0.272
*Prenatal risk factor*				
PROM, % (*n*)	25 (4)	25 (4)	/	0.657
Amniotic fluid pollution, %(*n*)	0 (0)	6.3 (1)	/	0.317
Intrauterine distress, % (*n*)	12.5 (2)	6.3 (1)	/	0.551
Maternal hypertension, %(*n*)	6.3 (1)	6.3 (1)	/	0.758
Gestational diabetes mellitus, %(*n*)	6.3 (1)	25 (4)	/	0.151
Antenatal steroid use, % (*n*)	87.5 (14)	62.5 (10)	/	0.108
*Risk factors before enrollment*				
Antibiotic course, *M* (P_25_–P_75_), *d*	4.5 (2.25–6.75)	4 (3–9.2)	−0.629	0.529
PN courses, *M* (P_25_–P_75_), *d*	8 (3.25–18.75)	19.50 (6.25–23.75)	−1.890	0.059
PICC period, *M* (P_25_–P_75_), d	8 (3.25–18.75)	16.50 (0.25–28.50)	−1.748	0.080
Formula milk feeding, % (*n*)	68.8 (11)	31.3 (5)	/	1.000
Duration of enrollment to diagnosis, *M* (P_25_-P_75_), *d*	/	4.00 (0.00, 11.5)	/	/
*Clinical manifestations when enrolled*				
Abdominal distention, %(*n*)	18.8 (3)	81.3 (3)	/	0.300
Vomiting, %(*n*)	87.5 (14)	12.5 (2)	/	0.197
Bloody Stool, %(*n*)	62.5 (10)	37.5 (6)	/	0.110
*Outcomes*				
Surgery, %(*n*)	0.0 (0)	25 (4)	/	0.109
Neonatal sepsis, % (*n*)	18.8 (3)	81.3 (13)	/	0.001
Shock, %(*n*)	6.3 (1)	18.8 (3)	/	0.293
hospital stays, *x* ± S. D, d	49.00 ± 26.71	76.94 ± 48.90	3.413	0.004

NEC, necrotizing enterocolitis; PROM, premature rupture of membranes > 18 h; PN, Parenteral nutrition; PICC, peripherally inserted central catheter.

### 3.2. Microbial information

To determine whether the sample size and sequences measured were sufficient and whether the grouping was meaningful, a core curve, rarefaction curve and analysis of similarities based on Bray–Curtis distance were performed. The core curve eventually plateaued, which showed that the size of the present study was reasonable (Supplementary Figure 1a), and the Shannon diversity index rarefaction curve showed that the sequences we measured were sufficient to reflect the diversity information (Supplementary Figure 1b). ANOSIM showed that the difference between the two groups was not significantly greater than that within the NEC and non-NEC groups, indicating that the grouping methods were meaningful (Supplementary Figure 1c).

The number of OTUs in the non-NEC and NEC groups was 1820 and 1935, respectively, and there were 958 OTUs shared by the two groups (Supplementary Figure 2a). At the phylum level, *Proteobacteria*, *Firmicutes*, *Actinomycetota* and *Bacteroidota* were the dominant phyla and accounted for more than 90% of the total abundance on the Circos diagram (Supplementary Figure 2b). At the genus level, *Enterobacter*, *Acinetobacter*, *Stenotrophomonas*, *Enterococcus*, *Escherichia-Shigella*, *Staphylococcus*, and *Klebsiella* were the main genera, and the abundances of different genera are shown in a heatmap with different colors (Supplementary Figure 2c). At the species level, the abundance is shown in the bar chart, which shows that the main species were from the dominant genera (Supplementary Figure 2d).

### 3.3. Diversity analysis

**Figure 2:**
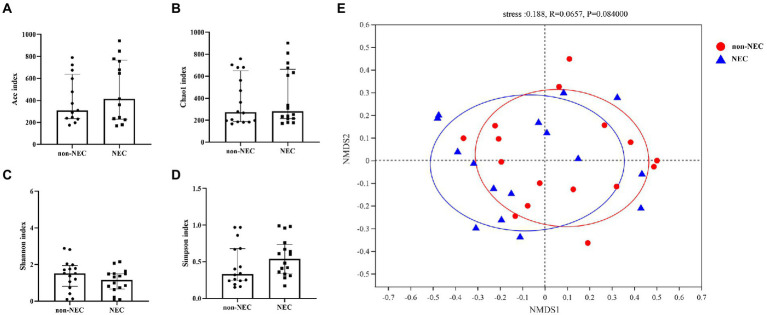
Alpha and beta diversity among the NEC and non-NEC groups. Alpha diversity indices, including Ace, Chao1, Shannon and Simpson indices, showed no differences between the NEC and non-NEC groups (*p* > 0.05). **(A–D)** There was no significant difference in the beta diversity between the two groups (*p* > 0.05) **(E).**

To explore the difference in richness and diversity between the two groups, diversities, including alpha and beta diversity, were compared first. The alpha diversity indices, including the Ace, Chao1, Shannon and Simpson indices, showed no differences between the two groups (*p* > 0.05, Figures 2A–D). NMDS showed that both samples of the NEC and non-NEC groups were discrete, and the beta diversity was also not significantly different between the two groups (*p* > 0.05, Figure 2E).

### 3.4. Microbiota characteristics

**Figure 3:**
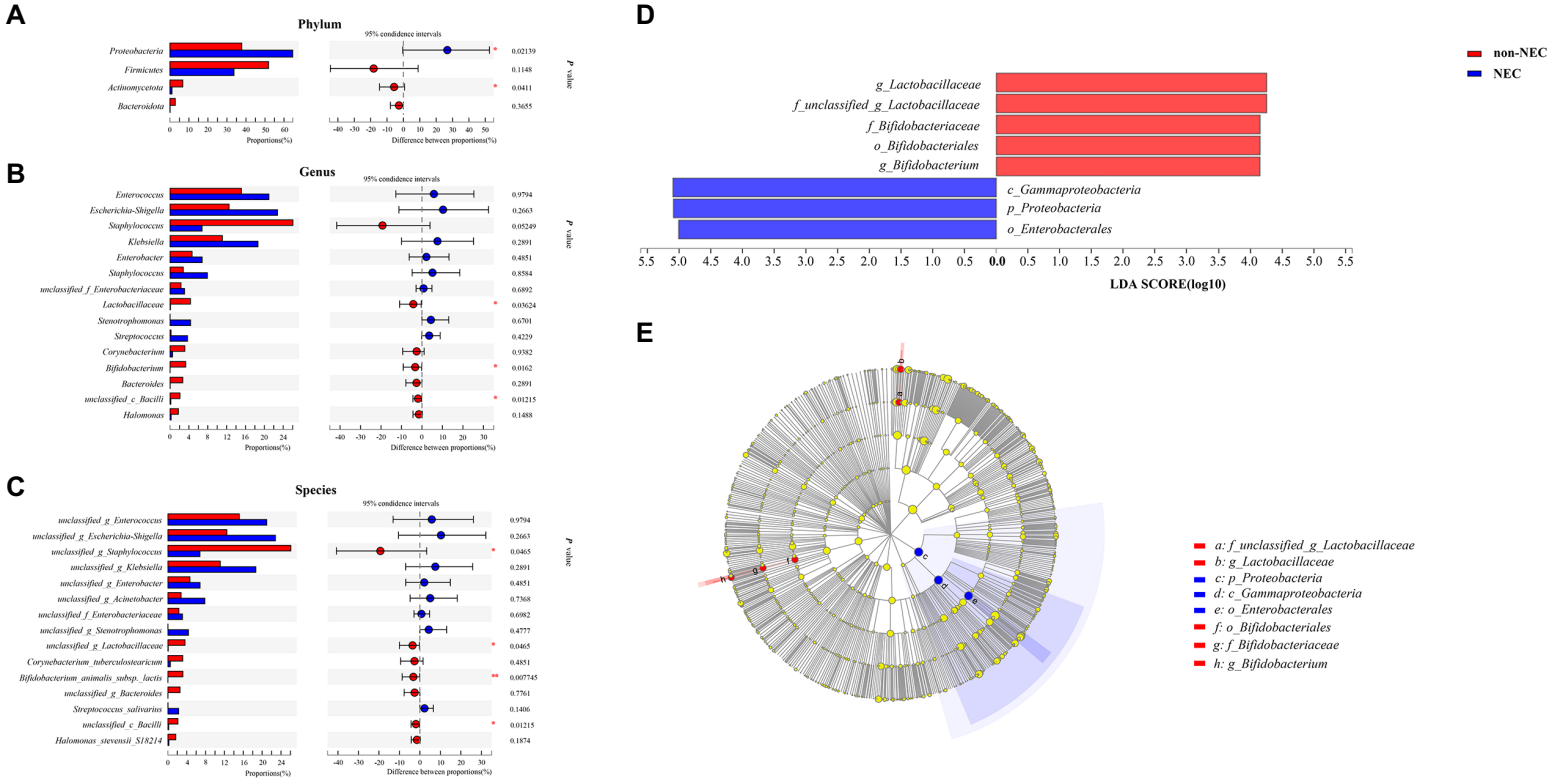
Community composition of the gut microbiota in the NEC and non-NEC groups. Differences between NEC and non-NEC groups at the phylum **(A)**, genus **(B)** and species **(C)** levels. Further *LEfSe* between the two groups **(D,E)**. *p < 0.05, **p < 0.01.

To determine the difference in microbiota composition between the non-NEC and NEC groups, we compared the microbiota at different levels. There were significant differences between the two groups at the phylum, genus and species levels. At the phylum level, *Proteobacteria* increased and *Actinomycetota* abundances decreased significantly in the NEC group compared to the non-NEC group (*p* < 0.05). *Firmicutes* and *Bacteroidota* showed no difference (*p* > 0.05) (Figure 3A). At the genus level, *Bifidobacterium, Lactobacillaceae* and *Bacilli* abundances decreased significantly in the NEC group (*p* < 0.05). *Enterococcus*, *Escherchia-Shigella*, *Staphylococcus*, *Klebsiella*, *Enterobacter* and *Acinetobacter* et al. showed no significant difference (*p* > 0.05) (Figure 3B). At the species level, unclassified *Staphylococcus, Lactobacillaceae, Bifidobacterium animalis subsp lactis* and *Bacilli* abundances decreased in the NEC group (*p* < 0.05, Figure 3C). To characterize the variation among microbiomes and to verify the effective value of these microbiota on the difference between groups, further *LEfSe* analysis showed the change in *Proteobacteria* abundance at the phylum level and *Lactobacillaceae* and *Bifidobacterium* abundances at the genus level scored higher than 4, indicating their relatively high significance when compared (*p* < 0.05, Figures 3D,E).

### 3.5. Metabolites measurement

**Figure 4:**
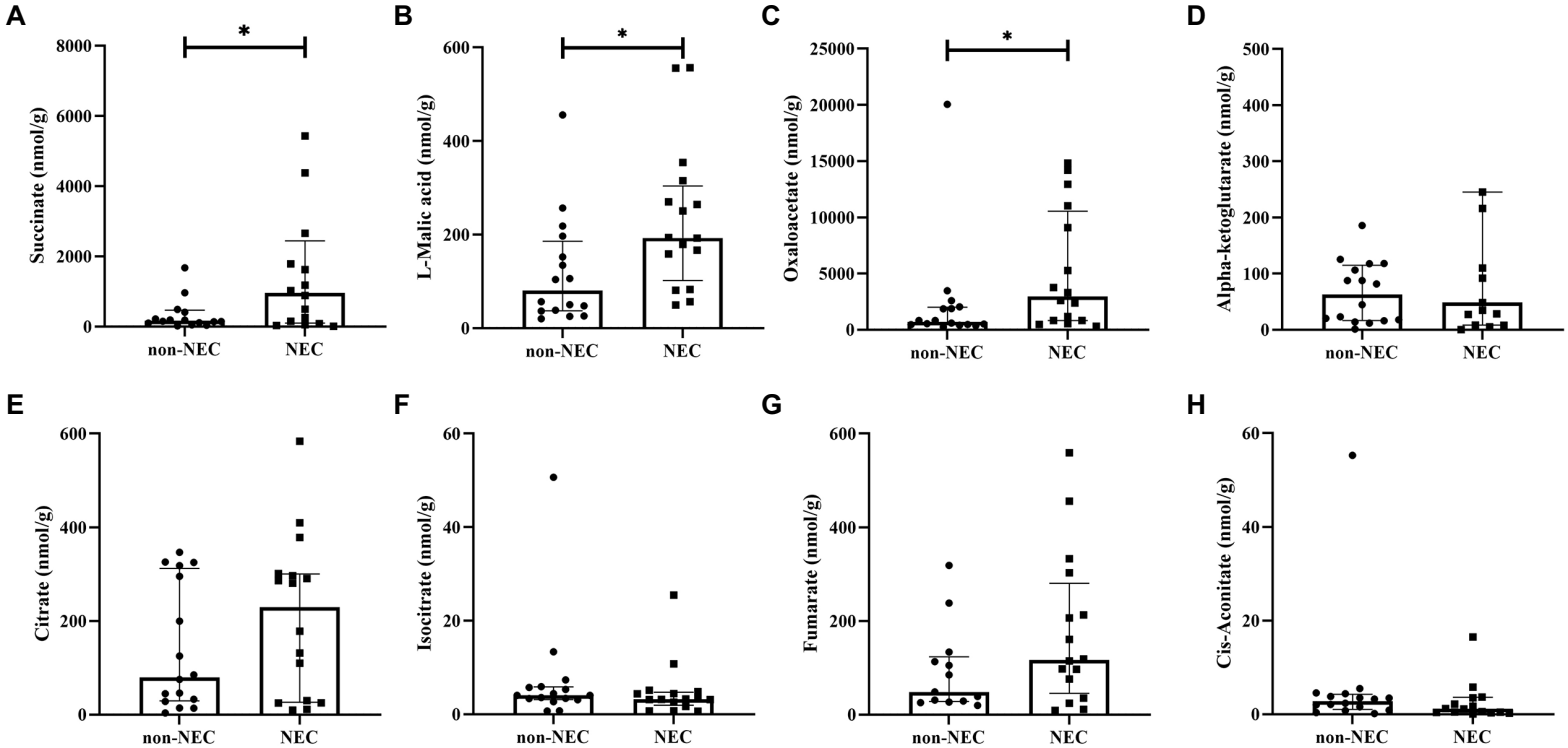
Comparison of TCA metabolites among the NEC and non-NEC groups. **(A)** succinate, **(B)** L-malic acid, **(C)** oxaloacetate, **(D)** alpha-ketoglutarate, **(E)** citrate, **(F)** isocitrate, **(G)** fumarate, and **(H)** cis-aconitate. *p < 0.05.

To determine the difference of metabolites between the two groups, the concentrations of TCA intermediates were analyzed, and we found that some of them were significantly different between the two groups. Succinate [956.42 (100.15, 2441.62) vs. 166.19 (101.61,466.87)], L-malic acid [193.05 (101.67, 303.94) vs. 80.33 (37.06, 185.60)] and oxaloacetate [2960.10 (826.28,10537.55) vs. 705.67 (452.56,1995.96)] concentrations were significantly increased in the NEC group compared to the non-NEC group (*p* < 0.05, Figures 4A–C). Moreover, citrate, cis-aconitate, isocitrate, alpha-ketoglutarate, and fumarate concentrations showed no statistically significant difference between the two groups (*p* > 0.05, Figures 4D–H).

**Figure 5:**
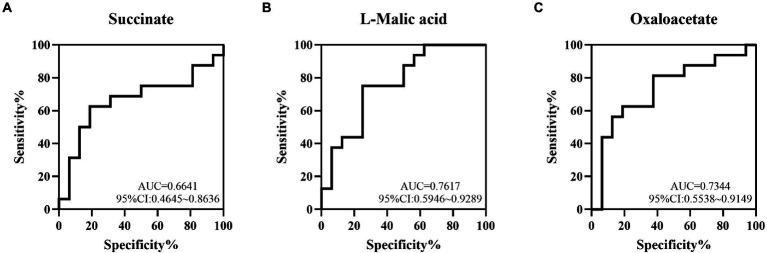
The value of some TCA metabolites in the early diagnosis of NEC by ROC curve analysis. The AUCs of succinate **(A)**, L-malic acid **(B)** and oxaloacetate **(C)** acids between the NEC and non-NEC groups were 0.6641, 0.7617 and 0.7344, respectively.

To determine the value of succinate, L-malic acid and oxaloacetate in predicting NEC, ROC analysis was performed, and the AUCs were 0.6641 (95%CI: 0.4645 ~ 0.8636), 0.7617 (95%CI: 0.5946 ~ 0.9289) and 0.7344 (95%CI: 0.5538 ~ 0.9149), respectively, which showed low-to-medium early diagnosis values (Figures 5A–C).

### 3.6. Relationship between metabolites and the gut microbiota

**Figure 6:**
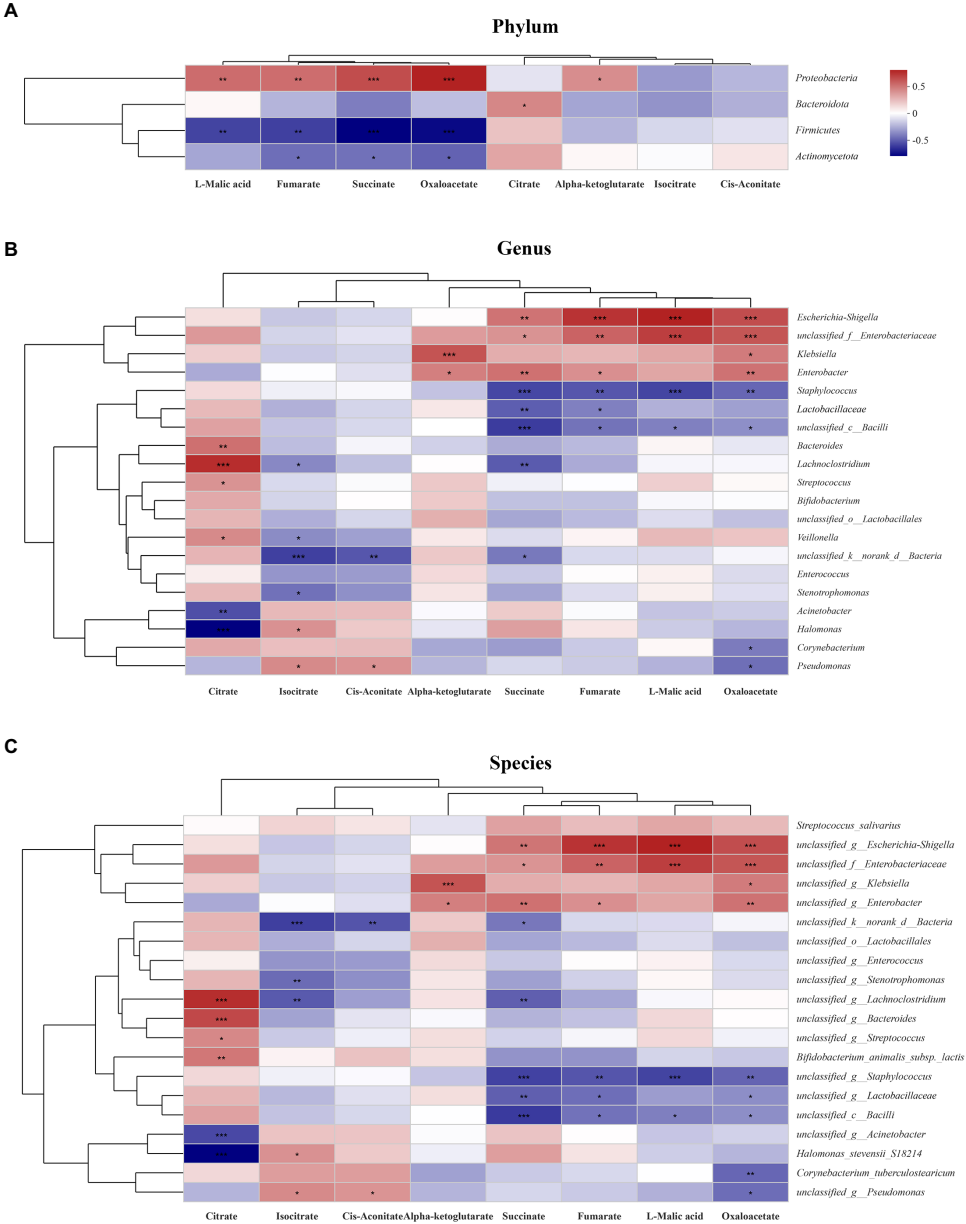
Relationship between the gut microbiota and TCA metabolites in the study. The relationship between the microbiota on phylum **(A)**, genus **(B)** and species **(C)** levels and the metabolites were showed in the heatmap. The change in colour reflects the data in the two-dimensional matrix. The colour depth indicates the size of the value, and it can intuitively express the size of the value in a defined colour depth.

To explore the relationship between gut microbiota and TCA metabolites, a heatmap was generated. At the phylum level, succinate, L-malic acid and oxaloacetate concentrations were positively correlated with *Proteobacteria* abundance and negatively correlated with *Firmicutes* and *Actinomycetota* abundances (Figure 6A). At the genus level, succinate, L-malic acid and oxaloacetate concentrations were positively correlated with *Escherichia coli-Shigella, Enterobacteriaceae*, and *Klebsiella* abundances and negatively correlated with *Staphylococcus*, *Lactobacillaceae*, and *unclassified bacilli* abundances (Figure 6B). At the species level, the results were consistent with those at the genus level (Figure 6C).

## 4. Discussion

Early diagnosis of NEC can be helpful to decrease mortality and improve outcomes. For infants with abdominal manifestations or signs but no typical NEC imaging presentations, early diagnosis is difficult, gut microbiota as well as its metabolites may help to early diagnose NEC. In this study, we found that increased *Proteobacteria* abundance and decreased *Lactobacillaceae* and *Bifidobacterium* abundances as well as increased succinate, L-malic acid and oxaloacetate concentrations before NEC development have potential early diagnostic value for NEC among infants with clinical manifestations.

We found that the *Proteobacteria* abundance increased and that of *Actinomycetota* decreased with significance at the phylum level. Moreover, at the genus level, *Bifidobacterium* and *Lactobacillaceae* abundances decreased significantly when infants were finally diagnosed with NEC. Consistent with our study, previous studies have shown that before the onset of NEC, the increase in *Proteobacteria* abundance and the decrease in *Firmicutes* and *Bacteroidota* abundances are the main features before NEC occurs (Pammi et al., 2017; Baranowski and Claud, 2019). *Klebsiella*, *Clostridium*, *Clostridium perfringens and Bacillota* have been reported increased (de la Cochetiere et al., 2004; Olm et al., 2019; Brehin et al., 2020) and *Propionibacterium*, *Bacteroidota*, *Lactobacillaceae, Pasteurella,* and *Parabacteroidota* decreased in NEC infants (Morrow et al., 2013; Fu et al., 2020). However, no specific bacteria have been indicated to be related to NEC. Thus, we further explored the microbiota biomarker deeply at the species level and found that *unclassified Staphylococcus*, *Lactobacillaceae* and *Bifidobacterium animalis subsp lactis* abundances decreased in NEC infants when they had clinical manifestations, which suggested that they have relatively high clinical value to help to early diagnose NEC in this study.

Accompanied by changes in the microbiota, metabolites also change in diseases, which are always considered the bridge between microbiota and host communication, and some metabolites may be used as biomarkers for the prediction of diseases (Ticinesi et al., 2019; Agus et al., 2021; Coker et al., 2022). Thus, based on the microbiota changes in NEC, we further explored the predictive value of TCA intermediates, which not only facilitate energy production and provide anabolic precursors but also function as intra- and extracellular metabolic signals regulating pleiotropic biological processes (Tong et al., 2022). To our knowledge, this is the first study to explore intermediates of the TCA cycle, including succinate, L-malic acid and oxaloacetate, as potential biomarkers, and they showed low to medium value for the prediction of NEC.

In our study, we found that the succinate concentration increased before NEC, and further correlation analysis showed that the succinate concentration was positively correlated with *Proteobacteria.* Succinate is a natural metabolic product in some bacteria, such as the *Veillonellaceae* and *Prevotellaceae* families (Connors et al., 2018; Serena et al., 2018), and is thought to be an inflammatory signal in the development of intestinal inflammation and fibrosis through its cognate receptor (Macias-Ceja et al., 2019). Succinate has been reported as a potential biomarker for predicting the incidence of overweight (Wan et al., 2020), aortic aneurysm and dissection (Cui et al., 2021), and cardiovascular disease (Serena et al., 2018). And in our study, we found that succinate might be a potential biomarker for early diagnosis of NEC.

Meanwhile, in our study, we found that increased L-malic acid and oxaloacetate might be potential biomarkers for early diagnosis of NEC. L-malic acid is formed by the hydration of fumarate and is oxidized into oxaloacetate in the last reaction of the TCA cycle (Louis et al., 2014). To date, little is known about the immunological roles of intracellular L-malic acid and oxaloacetate. Malic acid and oxaloacetate are presumed to be the metabolites mainly consumed by fungi, yeasts, and bacteria (Martino et al., 2018; Wei et al., 2021). Malic acid can be produced by *Saccharomyces* and *Aspergillus* (Pines et al., 1996; Li et al., 2021), and the production of oxaloacetate by the gut microbiota remains unknown. The contents of L-malic acid and oxaloacetate have been reported to change in diseases (Hou et al., 2017; Cabré et al., 2020; Morrow et al., 2022). L-malic acid can modulate blood pressure (Hou et al., 2017), and faecal L-malic acid levels were significantly reduced in gestational diabetes mellitus (Liang et al., 2019). The oxaloacetate concentration decreased in patients with severe obesity and non-alcoholic steatohepatitis (Cabré et al., 2020; Morrow et al., 2022). However, few studies have focused on the value of these compounds in intestinal diseases not mentioned in NEC. In our study, the correlation analysis showed that they were positively correlated with *Escherichia coli-Shigella*, *Enterobacteriaceae*, and *Klebsiella* abundances and negatively correlated with *Staphylococcus*, *Lactobacillaceae*, and *Unclassified Bacill* abundances, which have been reported to be changed in NEC infants (Morrow et al., 2013; McMurtry et al., 2015; Sim et al., 2015; Olm et al., 2019; Cuna et al., 2020). Thus, the increase in L-malic acid and oxaloacetate concentrations may reflect the microbiota change in NEC. Our ROC curve analysis found that malic acid and oxaloacetate had medium value for early diagnosis of NEC and further studies with larger sample size are needed for clinical application in the future.

### 4.1. Strengths and limitations

For infants without any clinical manifestations, it is easy to differentiate them from those NEC infants. However, it is difficult to make an early diagnosis whether they have NEC in infants just presenting with non-specific manifestations such as abdominal distention, vomiting, bloody stools and diarrhea. The strength of our studies is that all infants enrolled in this study all presented with one of those non-specific manifestations and we explored the value of gut microbiota and metabolite to early diagnose NEC which make it more valuable for clinical application. We first found that succinate, L-malic acid and oxaloacetate may be potential diagnosed biomarkers for the early diagnosis of NEC. However, there were still some limitations in our study. First, the sample size was small. Second, there was a lack of samples for the dynamic exploration of gut microbiota and metabolite changes when NEC was diagnosed. Therefore, further multicentre studies are necessary before the clinical application of gut microbiota and metabolites in the prediction of NEC.

## 5. Conclusion

Decreased *unclassified Staphylococcus*, *Lactobacillaceae* and *Bifidobacterium animalis subsp. lactis* abundances at the species level as well as the increase in the concentrations of some TCA metabolites, including succinate, L-malic acid and oxaloacetate, have potential value for the early diagnosis of NEC.

## Data availability statement

The microbiota databases for this study can be found in the NCBI (PRJNA871786: https://www.ncbi.nlm.nih.gov/sra/PRJNA871786), and other data can be obtained from the corresponding author.

## Ethics statement

The studies involving human participants were reviewed and approved by Ethics Committee of the Children’s Hospital of Chongqing Medical University. Written informed consent to participate in this study was provided by the participants’ legal guardian/next of kin.

## Author contributions

T-TD and X-CL collected the clinical data, processed the faecal samples, analyzed the data, and drafted the manuscript. XG and Z-ZL helped to collect the faecal samples. YH and L-QL supervised the project and contributed to the conception and design of the study and analysis and interpretation of the data. Z-LW and L-QL contributed to critical manuscript revisions. T-TD, X-CL, and L-QL provided the final approval of the manuscript. All authors contributed to the article and approved the submitted version.

## Funding

This work was supported by the Natural Science Foundation of Chongqing municipality (cstc2021jcyj-msxmX0063), Joint Medical Research Project of Chongqing Science and Technology Commission (2022MSXM039), Science and Health Project of Chongqing Health Commission (2020FYYX217), National Natural Science Foundation of China (82001602), China Postdoctoral Science Foundation (No. 2022MD713716), and the General Basic Research Project from the Ministry of Education Key Laboratory of Child Development and Disorders (No. GBRP-202107).

## Conflict of interest

The authors declare that the research was conducted in the absence of any commercial or financial relationships that could be construed as a potential conflict of interest.

## Publisher’s note

All claims expressed in this article are solely those of the authors and do not necessarily represent those of their affiliated organizations, or those of the publisher, the editors and the reviewers. Any product that may be evaluated in this article, or claim that may be made by its manufacturer, is not guaranteed or endorsed by the publisher.

## References

[ref1] Agus A. Clément K. Sokol H. (2021). Gut microbiota-derived metabolites as central regulators in metabolic disorders. Gut 70, 1174–1182. doi: 10.1136/gutjnl-2020-323071, PMID: 33272977 PMC8108286

[ref2] Akram M. (2014). Citric acid cycle and role of its intermediates in metabolism. Cell Biochem. Biophys. 68, 475–478. doi: 10.1007/s12013-013-9750-1 24068518

[ref3] Aziz M. Prince J. M. Wang P. (2022). Gut microbiome and necrotizing enterocolitis: understanding the connection to find a cure. Cell Host Microbe 30, 612–616. doi: 10.1016/j.chom.2022.04.003, PMID: 35550664

[ref4] Baranowski J. R. Claud E. C. (2019). Necrotizing Enterocolitis and the preterm infant microbiome. Adv. Exp. Med. Biol. 1125, 25–36. doi: 10.1007/5584_2018_313 30680646

[ref5] Berken J. A. Chang J. (2022). Neurologic consequences of neonatal necrotizing Enterocolitis. Dev. Neurosci. 44, 295–308. doi: 10.1159/000525378, PMID: 35697005

[ref6] Brehin C. Dubois D. Dicky O. Breinig S. Oswald E. Serino M. (2020). Evolution of gut microbiome and metabolome in suspected necrotizing Enterocolitis: a case-control study. J. Clin. Med. 9:2278. doi: 10.3390/jcm9072278, PMID: 32709038 PMC7408695

[ref7] Cabré N. Luciano-Mateo F. Baiges-Gayà G. Fernández-Arroyo S. Rodríguez-Tomàs E. Hernández-Aguilera A.. (2020). Plasma metabolic alterations in patients with severe obesity and non-alcoholic steatohepatitis. Aliment. Pharmacol. Ther. 51, 374–387. doi: 10.1111/apt.15606, PMID: 31825539

[ref8] Canfora E. E. Meex R. C. R. Venema K. Blaak E. E. (2019). Gut microbial metabolites in obesity, NAFLD and T2DM. Nat. Rev. Endocrinol. 15, 261–273. doi: 10.1038/s41574-019-0156-z, PMID: 30670819

[ref9] Cao Y. Jiang S. Sun J. Hei M. Wang L. Zhang H.. (2021). Assessment of neonatal intensive care unit practices, morbidity, and mortality among very preterm infants in China. JAMA Netw. Open 4:e2118904. doi: 10.1001/jamanetworkopen.2021.18904, PMID: 34338792 PMC8329742

[ref10] Cao X. Zhang L. Jiang S. Li M. Yan C. Shen C.. (2022). Epidemiology of necrotizing enterocolitis in preterm infants in China: a multicenter cohort study from 2015 to 2018. J. Pediatr. Surg. 57, 382–386. doi: 10.1016/j.jpedsurg.2021.05.014, PMID: 34175121

[ref11] Cassir N. Simeoni U. La Scola B. (2016). Gut microbiota and the pathogenesis of necrotizing enterocolitis in preterm neonates. Future Microbiol. 11, 273–292. doi: 10.2217/fmb.15.136 26855351

[ref12] Clarke K. R. (1993). Non-parametric multivariate analyses of changes in community structure. Aust. J. Ecol. 18, 117–143. doi: 10.1111/j.1442-9993.1993.tb00438.x

[ref13] Coker O. O. Liu C. Wu W. K. K. Wong S. H. Jia W. Sung J. J. Y.. (2022). Altered gut metabolites and microbiota interactions are implicated in colorectal carcinogenesis and can be non-invasive diagnostic biomarkers. Microbiome 10:35. doi: 10.1186/s40168-021-01208-5, PMID: 35189961 PMC8862353

[ref14] Connors J. Dawe N. Van Limbergen J. (2018). The role of succinate in the regulation of intestinal inflammation. Nutrients 11:25. doi: 10.3390/nu11010025, PMID: 30583500 PMC6356305

[ref15] Cui H. Chen Y. Li K. Zhan R. Zhao M. Xu Y.. (2021). Untargeted metabolomics identifies succinate as a biomarker and therapeutic target in aortic aneurysm and dissection. Eur. Heart J. 42, 4373–4385. doi: 10.1093/eurheartj/ehab605, PMID: 34534287 PMC11506060

[ref16] Cuna A. Yu W. Menden H. L. Feng L. Srinivasan P. Chavez-Bueno S.. (2020). NEC-like intestinal injury is ameliorated by lactobacillus rhamnosus GG in parallel with SIGIRR and A20 induction in neonatal mice. Pediatr. Res. 88, 546–555. doi: 10.1038/s41390-020-0797-6, PMID: 32053825 PMC8213439

[ref17] D'Apremont I. Marshall G. Musalem C. Mariani G. Musante G. Bancalari A.. (2020). Trends in perinatal practices and neonatal outcomes of very low birth weight infants during a 16-year period at NEOCOSUR centers. J. Pediatr. 225, 44–50.e1. doi: 10.1016/j.jpeds.2020.05.040, PMID: 32454113

[ref18] de la Cochetiere M.-F. Piloquet H. Des Robert C. Darmaun D. Galmiche J.-P. Roze J.-C. (2004). Early intestinal bacterial colonization and necrotizing enterocolitis in premature infants: the putative role of *Clostridium*. Pediatr. Res. 56, 366–370. doi: 10.1203/01.PDR.0000134251.45878.D5, PMID: 15201403

[ref19] Di Segni A. Braun T. BenShoshan M. Farage Barhom S. Glick Saar E. Cesarkas K.. (2018). Guided protocol for fecal microbial characterization by 16S rRNA-amplicon sequencing. J. Vis. Exp. 133:56845. doi: 10.3791/56845, PMID: 29608151 PMC5933208

[ref20] Dong L.-N. Wang M. Guo J. Wang J.-P. (2019). Role of intestinal microbiota and metabolites in inflammatory bowel disease. Chin. Med. J. 132, 1610–1614. doi: 10.1097/CM9.0000000000000290, PMID: 31090547 PMC6616233

[ref21] Eaton S. (2017). Necrotizing enterocolitis symposium: epidemiology and early diagnosis. J. Pediatr. Surg. 52, 223–225. doi: 10.1016/j.jpedsurg.2016.11.013, PMID: 27914586

[ref22] Frost B. L. Modi B. P. Jaksic T. Caplan M. S. (2017). New medical and surgical insights into neonatal necrotizing *Enterocolitis*: a review. JAMA Pediatr. 171, 83–88. doi: 10.1001/jamapediatrics.2016.2708, PMID: 27893069

[ref23] Fu C.-Y. Li L.-Q. Yang T. She X. Ai Q. Wang Z.-L. (2020). Autoinducer-2 may be a new biomarker for monitoring neonatal necrotizing *Enterocolitis*. Front. Cell. Infect. Microbiol. 10:140. doi: 10.3389/fcimb.2020.00140, PMID: 32373545 PMC7179697

[ref24] Geng Q. Wang Y. Li L. Guo C. (2018). Early postoperative outcomes of surgery for intestinal perforation in NEC based on intestinal location of disease. Medicine 97:e12234. doi: 10.1097/MD.0000000000012234, PMID: 30278493 PMC6181543

[ref25] Han J. Jackson D. Holm J. Turner K. Ashcraft P. Wang X.. (2018). Elevated d-2-hydroxyglutarate during colitis drives progression to colorectal cancer. Proc. Natl. Acad. Sci. U. S. A. 115, 1057–1062. doi: 10.1073/pnas.1712625115, PMID: 29339485 PMC5798335

[ref26] Hou E. Sun N. Zhang F. Zhao C. Usa K. Liang M.. (2017). Malate and aspartate increase L-arginine and nitric oxide and attenuate hypertension. Cell Rep. 19, 1631–1639. doi: 10.1016/j.celrep.2017.04.071, PMID: 28538181

[ref27] Karu N. Deng L. Slae M. Guo A. C. Sajed T. Huynh H.. (2018). A review on human fecal metabolomics: methods, applications and the human fecal metabolome database. Anal. Chim. Acta 1030, 1–24. doi: 10.1016/j.aca.2018.05.031, PMID: 30032758

[ref28] Kliegman R. M. Walsh M. C. (1987). Neonatal necrotizing enterocolitis: pathogenesis, classification, and spectrum of illness. Curr. Probl. Pediatr. 17, 213–288. doi: 10.1016/0045-9380(87)90031-4, PMID: 3556038 PMC7130819

[ref29] Kovatcheva-Datchary P. Nilsson A. Akrami R. Lee Y. S. De Vadder F. Arora T.. (2015). Dietary fiber-induced improvement in glucose metabolism is associated with increased abundance of *Prevotella*. Cell Metab. 22, 971–982. doi: 10.1016/j.cmet.2015.10.001, PMID: 26552345

[ref30] Li S.-F. Zhang S.-B. Lv Y.-Y. Zhai H.-C. Li N. Hu Y.-S.. (2021). Metabolomic analyses revealed multifaceted effects of hexanal on *Aspergillus flavus* growth. Appl. Microbiol. Biotechnol. 105, 3745–3757. doi: 10.1007/s00253-021-11293-z, PMID: 33880599

[ref31] Liang S. Hou Z. Li X. Wang J. Cai L. Zhang R.. (2019). The fecal metabolome is associated with gestational diabetes mellitus. RSC Adv. 9, 29973–29979. doi: 10.1039/c9ra05569j, PMID: 35531557 PMC9072113

[ref32] Liu X.-C. Du T.-T. Gao X. Zhao W.-J. Wang Z.-L. He Y.. (2022). Gut microbiota and short-chain fatty acids may be new biomarkers for predicting neonatal necrotizing enterocolitis: a pilot study. Front. Microbiol. 13:969656. doi: 10.3389/fmicb.2022.969656, PMID: 36060739 PMC9428482

[ref33] Louis P. Flint H. J. (2017). Formation of propionate and butyrate by the human colonic microbiota. Environ. Microbiol. 19, 29–41. doi: 10.1111/1462-2920.13589 27928878

[ref34] Louis P. Hold G. L. Flint H. J. (2014). The gut microbiota, bacterial metabolites and colorectal cancer. Nat. Rev. Microbiol. 12, 661–672. doi: 10.1038/nrmicro3344 25198138

[ref35] Macia L. Tan J. Vieira A. T. Leach K. Stanley D. Luong S.. (2015). Metabolite-sensing receptors GPR43 and GPR109A facilitate dietary fibre-induced gut homeostasis through regulation of the inflammasome. Nat. Commun. 6:6734. doi: 10.1038/ncomms7734, PMID: 25828455

[ref36] Macias-Ceja D. C. Ortiz-Masiá D. Salvador P. Gisbert-Ferrándiz L. Hernández C. Hausmann M.. (2019). Succinate receptor mediates intestinal inflammation and fibrosis. Mucosal Immunol. 12, 178–187. doi: 10.1038/s41385-018-0087-3, PMID: 30279517

[ref37] Martino G. P. Perez C. E. Magni C. Blancato V. S. (2018). Implications of the expression of enterococcus faecalis citrate fermentation genes during infection. PLoS One 13:e0205787. doi: 10.1371/journal.pone.0205787, PMID: 30335810 PMC6193673

[ref38] McCarville J. L. Chen G. Y. Cuevas V. D. Troha K. Ayres J. S. (2020). Microbiota metabolites in health and disease. Annu. Rev. Immunol. 38, 147–170. doi: 10.1146/annurev-immunol-071219-125715 32340573

[ref39] McMurtry V. E. Gupta R. W. Tran L. Blanchard E. E. Penn D. Taylor C. M.. (2015). Bacterial diversity and clostridia abundance decrease with increasing severity of necrotizing enterocolitis. Microbiome 3:11. doi: 10.1186/s40168-015-0075-8, PMID: 25810906 PMC4373520

[ref40] Meister A. L. Doheny K. K. Travagli R. A. (2020). Necrotizing enterocolitis: It's not all in the gut. Exp. Biol. Med. (Maywood) 245, 85–95. doi: 10.1177/1535370219891971, PMID: 31810384 PMC7016421

[ref41] Morrow M. R. Batchuluun B. Wu J. Ahmadi E. Leroux J. M. Mohammadi-Shemirani P.. (2022). Inhibition of ATP-citrate lyase improves NASH, liver fibrosis, and dyslipidemia. Cell Metab. 34, 919–936.e8. doi: 10.1016/j.cmet.2022.05.004, PMID: 35675800

[ref42] Morrow A. L. Lagomarcino A. J. Schibler K. R. Taft D. H. Yu Z. Wang B.. (2013). Early microbial and metabolomic signatures predict later onset of necrotizing enterocolitis in preterm infants. Microbiome 1:13. doi: 10.1186/2049-2618-1-13, PMID: 24450576 PMC3971624

[ref43] Neu J. (2020). Necrotizing Enterocolitis: the future. Neonatology 117, 240–244. doi: 10.1159/000506866 32155645

[ref44] Ng P. C. (2018). An update on biomarkers of necrotizing enterocolitis. Semin. Fetal Neonatal Med. 23, 380–386. doi: 10.1016/j.siny.2018.07.006, PMID: 30082194

[ref45] Nicholson J. K. Holmes E. Kinross J. Burcelin R. Gibson G. Jia W.. (2012). Host-gut microbiota metabolic interactions. Science 336, 1262–1267. doi: 10.1126/science.1223813 22674330

[ref46] Olm M. R. Bhattacharya N. Crits-Christoph A. Firek B. A. Baker R. Song Y. S.. (2019). Necrotizing enterocolitis is preceded by increased gut bacterial replication, *Klebsiella*, and fimbriae-encoding bacteria. Sci. Adv. 5:eaax5727. doi: 10.1126/sciadv.aax5727, PMID: 31844663 PMC6905865

[ref47] Pammi M. Cope J. Tarr P. I. Warner B. B. Morrow A. L. Mai V.. (2017). Intestinal dysbiosis in preterm infants preceding necrotizing enterocolitis: a systematic review and meta-analysis. Microbiome 5:31. doi: 10.1186/s40168-017-0248-8, PMID: 28274256 PMC5343300

[ref48] Pines O. Even-Ram S. Elnathan N. Battat E. Aharonov O. Gibson D.. (1996). The cytosolic pathway of L-malic acid synthesis in *Saccharomyces cerevisiae*: the role of fumarase. Appl. Microbiol. Biotechnol. 46, 393–399. doi: 10.1007/BF00166235, PMID: 8987728

[ref49] Qi X. Yun C. Sun L. Xia J. Wu Q. Wang Y.. (2019). Gut microbiota-bile acid-interleukin-22 axis orchestrates polycystic ovary syndrome. Nat. Med. 25, 1225–1233. doi: 10.1038/s41591-019-0509-0, PMID: 31332392 PMC7376369

[ref50] Quast C. Pruesse E. Yilmaz P. Gerken J. Schweer T. Yarza P.. (2013). The SILVA ribosomal RNA gene database project: improved data processing and web-based tools. Nucleic Acids Res. 41, D590–D596. doi: 10.1093/nar/gks1219, PMID: 23193283 PMC3531112

[ref51] Roca M. Alcoriza M. I. Garcia-Cañaveras J. C. Lahoz A. (2021). Reviewing the metabolome coverage provided by LC-MS: focus on sample preparation and chromatography-A tutorial. Anal. Chim. Acta 1147, 38–55. doi: 10.1016/j.aca.2020.12.025, PMID: 33485584

[ref52] Rognes T. Flouri T. Nichols B. Quince C. Mahé F. (2016). VSEARCH: a versatile open source tool for metagenomics. PeerJ 4:e2584. doi: 10.7717/peerj.2584, PMID: 27781170 PMC5075697

[ref53] Segata N. Izard J. Waldron L. Gevers D. Miropolsky L. Garrett W. S.. (2011). Metagenomic biomarker discovery and explanation. Genome Biol. 12:R60. doi: 10.1186/gb-2011-12-6-r60, PMID: 21702898 PMC3218848

[ref54] Serena C. Ceperuelo-Mallafré V. Keiran N. Queipo-Ortuño M. I. Bernal R. Gomez-Huelgas R.. (2018). Elevated circulating levels of succinate in human obesity are linked to specific gut microbiota. ISME J. 12, 1642–1657. doi: 10.1038/s41396-018-0068-2, PMID: 29434314 PMC6018807

[ref55] Shahi S. K. Zarei K. Guseva N. V. Mangalam A. K. (2019). Microbiota analysis using two-step PCR and next-generation 16S rRNA gene sequencing. J. Vis. Exp. 152. doi: 10.3791/59980 PMC6945761 31680682

[ref56] Sharma R. Hudak M. L. (2013). A clinical perspective of necrotizing enterocolitis: past, present, and future. Clin. Perinatol. 40, 27–51. doi: 10.1016/j.clp.2012.12.012, PMID: 23415262 PMC3575605

[ref57] Sharon G. Garg N. Debelius J. Knight R. Dorrestein P. C. Mazmanian S. K. (2014). Specialized metabolites from the microbiome in health and disease. Cell Metab. 20, 719–730. doi: 10.1016/j.cmet.2014.10.016, PMID: 25440054 PMC4337795

[ref58] Sim K. Shaw A. G. Randell P. Cox M. J. McClure Z. E. Li M.-S.. (2015). Dysbiosis anticipating necrotizing enterocolitis in very premature infants. Clin. Infect. Dis. 60, 389–397. doi: 10.1093/cid/ciu822, PMID: 25344536 PMC4415053

[ref59] Ticinesi A. Nouvenne A. Tana C. Prati B. Meschi T. (2019). Gut microbiota and microbiota-related metabolites as possible biomarkers of cognitive aging. Adv. Exp. Med. Biol. 1178, 129–154. doi: 10.1007/978-3-030-25650-0_8, PMID: 31493226

[ref60] Tong W. Hannou S. A. Wang Y. Astapova I. Sargsyan A. Monn R.. (2022). The intestine is a major contributor to circulating succinate in mice. FASEB J. 36:e22546. doi: 10.1096/fj.202200135RR, PMID: 36106538 PMC9523828

[ref61] Wan Y. Yuan J. Li J. Li H. Yin K. Wang F.. (2020). Overweight and underweight status are linked to specific gut microbiota and intestinal tricarboxylic acid cycle intermediates. Clin. Nutrit. 39, 3189–3198. doi: 10.1016/j.clnu.2020.02.014, PMID: 32164980

[ref62] Warner B. B. Tarr P. I. (2016). Necrotizing enterocolitis and preterm infant gut bacteria. Semin. Fetal Neonatal Med. 21, 394–399. doi: 10.1016/j.siny.2016.06.001, PMID: 27343151 PMC5116248

[ref63] Wei Z. Xu Y. Xu Q. Cao W. Huang H. Liu H. (2021). Microbial biosynthesis of L-malic acid and related metabolic engineering strategies: advances and prospects. Front. Bioeng. Biotechnol. 9:765685. doi: 10.3389/fbioe.2021.765685, PMID: 34660563 PMC8511312

[ref64] Xie B. Zu X. Wang Z. Xu X. Liu G. Liu R. (2022). Ginsenoside Rc ameliorated atherosclerosis regulating gut microbiota and fecal metabolites. Front. Pharmacol. 13:990476. doi: 10.3389/fphar.2022.990476, PMID: 36188559 PMC9520581

[ref65] Xu J. Bjursell M. K. Himrod J. Deng S. Carmichael L. K. Chiang H. C.. (2003). A genomic view of the human-Bacteroides thetaiotaomicron symbiosis. Science 299(5615, 2074–2076. doi: 10.1126/science.1080029 12663928

[ref66] Xu Y. Zhu X. Wang H. Pan Z. Li X. Guo X.. (2022). Prevalence of major morbidities and outcome of all hospitalized neonates. A retrospective cohort study of Huai'an neonatal survivals. J. Matern. Fetal Neonatal Med. 35, 9800–9810. doi: 10.1080/14767058.2022.2054320, PMID: 35341440

[ref67] Yan Z. Yang F. Sun L. Yu J. Sun L. Si Y.. (2022). Role of gut microbiota-derived branched-chain amino acids in the pathogenesis of Parkinson's disease: an animal study. Brain Behav. Immun. 106, 307–321. doi: 10.1016/j.bbi.2022.09.009, PMID: 36126853

[ref68] Yu L. Tian J. Zhao X. Cheng P. Chen X. Yu Y.. (2016). Bowel perforation in premature infants with necrotizing *Enterocolitis*: risk factors and outcomes. Gastroenterol. Res. Pract. 2016, 6134187–6134186. doi: 10.1155/2016/6134187, PMID: 27375739 PMC4916290

[ref69] Zhang H. Yu X. Zhang Z. Liu Z. Tang C. Zhao K.. (2020). Nanoliter-scale next-generation sequencing library-mediated high-throughput 16S rRNA microbial community profiling. BioTechniques 68, 204–210. doi: 10.2144/btn-2019-0102, PMID: 32096668

[ref70] Zhu W. Gregory J. C. Org E. Buffa J. A. Gupta N. Wang Z.. (2016). Gut microbial metabolite TMAO enhances platelet hyperreactivity and thrombosis risk. Cells 165, 111–124. doi: 10.1016/j.cell.2016.02.011, PMID: 26972052 PMC4862743

