# Supplementary material for: Changes of gut microbiota and tricarboxylic acid metabolites may be helpful in early diagnosis of necrotizing enterocolitis: A pilot study
Source: Front Microbiol. 2023 Mar 15;14:1119981. doi: 10.3389/fmicb.2023.1119981 (PMC10050441; doi:10.3389/fmicb.2023.1119981)
Supplement: Supplementary file 1 [file Data_Sheet_1.PDF]

# *Supplementary Material*

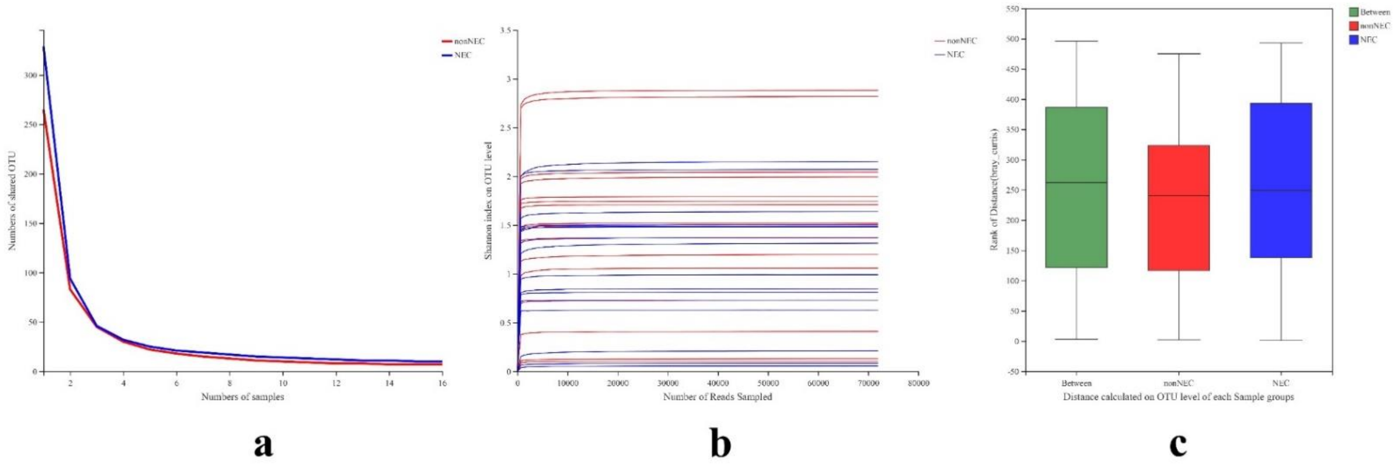


**Suppl. 1 Verification of the sample size, grouping and sequences.** *Core* analysis showed that the curve eventually flattened, and the sample size was reasonable. **(a)**. The flat rarefaction curve showed that the numbers of sequences measured were enough to reflect the diversity information. **(b)** Analysis of similarities (ANOSIM) showed that the difference among the two groups was not significantly greater than that within the groups, which means that the three groups were comparable **(c).**

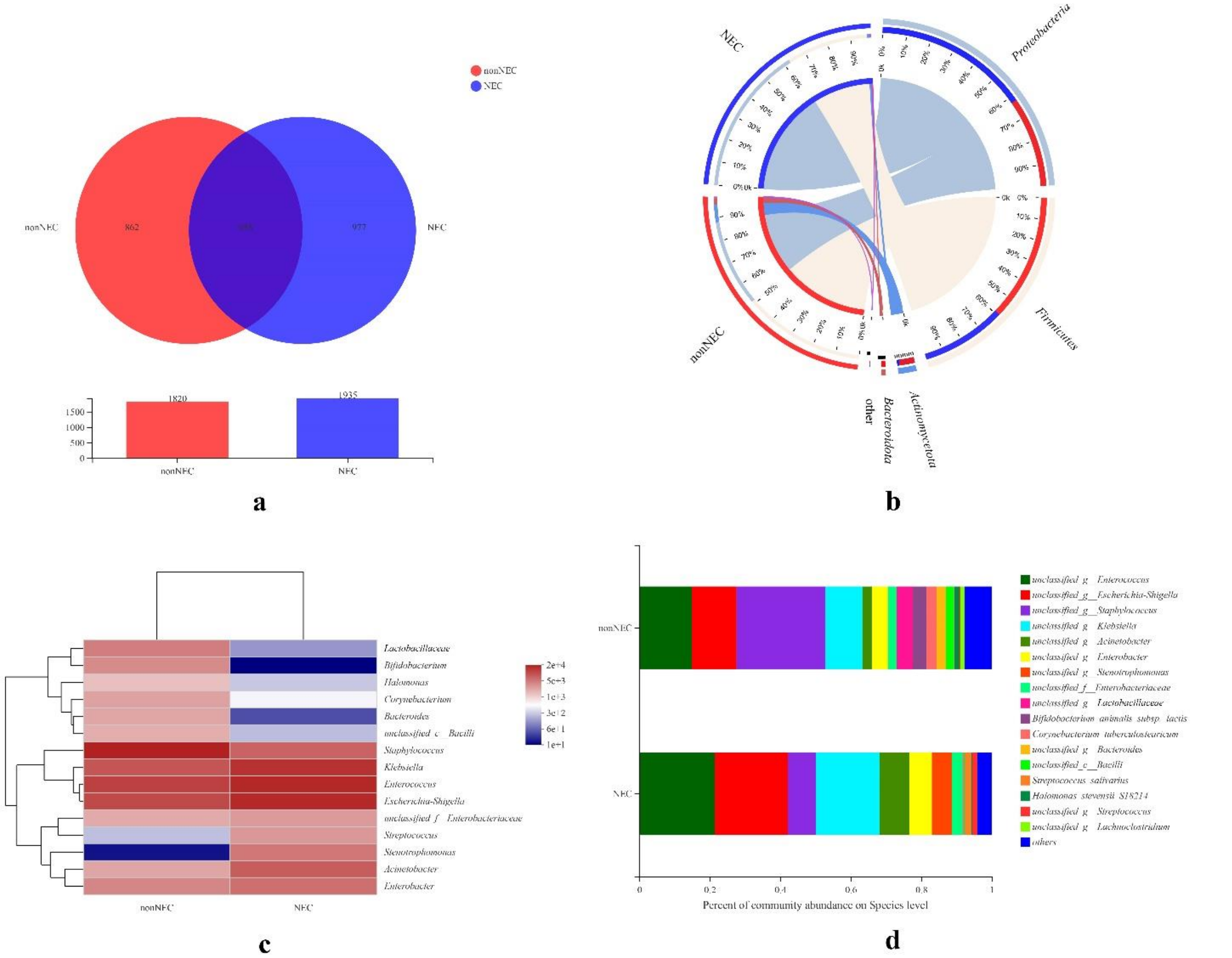


**Suppl. 2 Composition of the microbiota on different levels.** Venn diagrams showing the numbers and shared relationships of OTUs in the NEC and non-NEC groups **(a).** At the phylum level, *Proteobacteria*, *Firmicutes*, *Actinomycetota* and *Bacteroidota* were the dominant phyla **(b).** At the genus level, *Enterobacter*, *Acinetobacter*, *Stenotrophomonas*, *Enterococcus*, *Escherichia-Shigella*, *Staphylococcus*, and *Klebsiella* were the main genera **(c).** At the species level, the abundance is shown in the bar chart, which shows that the main species were from the dominant genera **(d).**
